# Supplementary material for: Inference in High-Dimensional Online Changepoint Detection
Source: J Am Stat Assoc. 2023 May 26;119(546):1461–72. doi: 10.1080/01621459.2023.2199962 (PMC11225951; doi:10.1080/01621459.2023.2199962)
Supplement: Supplemental Material [file UASA_A_2199962_SM5704.zip › Supp/acc-form.pdf]

Author Contributions Checklist Form

This form documents the artifacts associated with the article (i.e., the data and code supporting the computational findings) and describes how to reproduce the findings.

Part 1: Data

☐ This paper **does not** involve analysis of external data (i.e., no data are used or the only data are generated by the authors via simulation in their code).

☒ I certify that the author(s) of the manuscript have legitimate access to and permission to use the data used in this manuscript.

Abstract

We use a dataset of weekly deaths in the United States between January 2017 and June 2020 (available at: https://www.cdc.gov/nchs/nvss/vsrr/covid19/excess_deaths.htm). There are 12506 rows (excl. headers) and 12 columns in this dataset.

Availability

☒ Data **are** publicly available
☐ Data **cannot be made** publicly available

If the data are publicly available, see the *Publicly available data* section. Otherwise, see the *Non-publicly available dat*a section, below.

Publicly available data

☐ Data are available online at:
☒ Data are available as part of the paper's supplementary material.
☐ Data are publicly available by request, following the process described here:

☐ Data are or will be made available through some other mechanism, described here:

Non-publicly available data

Discussion of lack of publicly available data:

Description

File format(s)

☒ CSV or other plain text: US_weekly_deaths.csv
☐ Software-specific binary format (.Rda, Python pickle, etc.):
☐ Standardized binary format (e.g., netCDF, HDF5, etc.):
☐ Other (described here):

Data dictionary

☒ Provided by the authors in the following file(s): README.md
☐ Data file(s) is (are) self-describiing (e.g., netCDF files)
☐ Available at the following URL:

Additional information (optional)

Part 2: Code

Abstract

The codes implement the ocd_CI algorithm described in the submitted manuscript. In the main algorithm, we output a confidence interval for the changepoint location and estimate the set of indices of coordinates in which the mean changes, all under online changepoint monitoring.

Description

Code format(s)

☒ Script files
- ☒ R ☐ Python ☐ Matlab
- ☐ Other:

☐ Package
- ☐ R ☐ Python ☐ MATLAB toolbox
- ☐ Other:

☐ Reproducible report
- ☐ R Markdown ☐ Jupyter notebook
- ☐ Other:

☐ Shell script

☐ Other (described here):

Supporting software requirements

Version of primary software used

R version 3.6.2.

Libraries and dependencies used by the code

MASS 7.3-55

Supporting system/hardware requirements (optional)

Parallelization used

☒ No parallel code used
☐ Multi-core parallelization on a single machine/node
Number of cores used:
☐ Multi-machine/multi-node parallelization
Number of nodes and cores used:

License

☒ MIT License (default)
☐ BSD
☐ GPL v3.0
☐ Creative Commons
☐ Other (described here):

Additional information (optional)

Part 3: Reproducibility workflow

Scope

The provided workflow reproduces:
☒ Any numbers provided in text in the paper
☒ The computational method(s) presented in the paper (i.e., code is provided that implements the method(s))
☒ All tables and figures in the paper
☐ Selected tables and figures in the paper, as explained and justified here:

Workflow details

Format(s)

☒ Single master code file
☐ Wrapper (shell) script(s)
☐ Self-contained R Markdown file, Jupyter notebook, or other literate programming approach
☒ Text file (e.g., a readme-style file) that documents workflow
☐ Makefile
☐ Other (more detail in 'Instructions' below)

Instructions

ocd_CI.R is the main function which implements the proposed method.
table.R reproduces all tables and figures (apart from the real data Figure 3).
US_excess_deaths.R provides codes for the application on the real dataset and produces Figure 3.
All above information can be found in README.md.

Expected run-time

Approximate time needed to reproduce the analyses on a standard desktop machine:
☐ <1 minute
☐ 1-10 minutes

☐ 10-60 minutes
☐ 1-8 hours
☒ >8 hours
☐ Not feasible to run on a desktop machine, as described here:

Additional documentation (optional)

Notes (optional)
