# Supplementary material for: Inference in High-Dimensional Online Changepoint Detection
Source: J Am Stat Assoc. 2023 May 26;119(546):1461–72. doi: 10.1080/01621459.2023.2199962 (PMC11225951; doi:10.1080/01621459.2023.2199962)
Supplement: Supplemental Material [file UASA_A_2199962_SM5704.zip › Supp/Change_CI_supp_final.pdf]

Supplement to "Inference in High-dimensional Online Changepoint Detection"

Yudong Chen[*,†], Tengyao Wang[†], Richard J. Samworth[*]

[*] Statistical Laboratory, University of Cambridge
Wilberforce Road, Cambridge, CB3 0WB, United Kingdom
[†] Department of Statistics, London School of Economics
Houghton Street, London, WC2A 2AE, United Kingdom
Contact: t.wang59@lse.ac.uk

In this supplementary material, we provide proofs of the results stated in the main text (Section S1), auxiliary results and their proofs (Section S2), pseudocode for the `ocd` and `ocd′` base procedures (Section S3), extensions of our results to sub-Gaussian and sub-exponential settings (Section S4) and additional simulation results (Section S5). Throughout this supplement, we use $\mathbb{P}$ instead of $\mathbb{P}_{z,\theta,\Sigma}$ when it is clear from the context.

S1. PROOFS OF MAIN RESULTS

Proof of Theorem 1. Fix $r \geq 1$ that satisfies the assumption (3) in the theorem, $n > z$, $j \in [p]$, $b \in \mathcal{B}$ and $j' \in [p] \setminus \{j\}$. We assume, without loss of generality, that $\theta^{j'} \geq 0$. The case $\theta^{j'} < 0$ can be analyzed similarly. Recall that $b_{\min}$, defined in Algorithm 1, is the smallest positive scale in $\mathcal{B} \cup \mathcal{B}_0$, and write $b_{\mathrm{aux}}^{j'} := \min\bigl\{b \in (\mathcal{B} \cup \mathcal{B}_0) \cap (0, \infty) : b \geq \theta^{j'}\bigr\}$. Then we

have $A^{j',j}_{n,b} + \sum_{i=n+1}^{n+\ell} X_i^{j'} \mid t^j_{n,b} \sim \mathcal{N}\big(\theta^{j'} \min\{n+\ell-z, t^j_{n,b}+\ell\},\, t^j_{n,b}+\ell\big)$. Thus, recalling the definition of $\hat{\mathcal{S}}$ and $\tilde{b}^{j'}$ from Algorithm 1, we have

$$\begin{aligned}
&\mathbb{P}\big(\{j' \in \hat{\mathcal{S}}\} \cap \{\tilde{b}^{j'} \notin (0, \theta^{j'})\} \cap \{N = n, \hat{j} = j, \hat{b} = b\}\big) \\
&\qquad = \mathbb{E}\Big\{\mathbb{P}\Big(\{j' \in \hat{\mathcal{S}}\} \cap \{\tilde{b}^{j'} \notin (-b_{\min}, b^{j'}_{\mathrm{aux}})\} \cap \{N = n, \hat{j} = j, \hat{b} = b\} \;\Big|\; t^j_{n,b}\Big)\Big\} \\
&\qquad \le \mathbb{E}\bigg\{\mathbb{P}\bigg(A^{j',j}_{n,b} + \sum_{i=n+1}^{n+\ell} X_i^{j'} \ge b^{j'}_{\mathrm{aux}}(t^j_{n,b}+\ell) + d_1\big(t^j_{n,b}+\ell\big)^{1/2} \;\bigg|\; t^j_{n,b}\bigg)\bigg\} \\
&\qquad\qquad + \mathbb{E}\bigg\{\mathbb{P}\bigg(A^{j',j}_{n,b} + \sum_{i=n+1}^{n+\ell} X_i^{j'} \le -b_{\min}(t^j_{n,b}+\ell) - d_1\big(t^j_{n,b}+\ell\big)^{1/2} \;\bigg|\; t^j_{n,b}\bigg)\bigg\} \\
&\qquad \le \mathbb{E}\big\{\bar{\Phi}\big((b^{j'}_{\mathrm{aux}} - \theta^{j'})(t^j_{n,b}+\ell)^{1/2} + d_1\big)\big\} + \mathbb{E}\big\{\bar{\Phi}\big((b_{\min} + \theta^{j'})(t^j_{n,b}+\ell)^{1/2} + d_1\big)\big\} \\
&\qquad \le 2\bar{\Phi}(d_1).
\end{aligned} \tag{S1}$$

Moreover, by a similar argument to (S22) in the proof of Proposition S1, for $b \in (0, \theta^{j'})$, we have

$$\mathbb{P}\big(n - t^{j'}_{n,b} - d_2/b^2 > z\big) \le 2\bar{\Phi}\bigg(\frac{\sqrt{d_2}}{b}(\theta^{j'} - b/2)\bigg) \le 2\bar{\Phi}\big(\sqrt{d_2}/2\big). \tag{S2}$$

Combining (S1) and (S2), we have

$$\begin{aligned}
&\mathbb{P}\Big(\{j' \in \hat{\mathcal{S}}\} \cap \{n - t^{j'}_{n,\tilde{b}^{j'}} - d_2/(\tilde{b}^{j'})^2 > z\} \cap \{N = n, \hat{j} = j, \hat{b} = b\}\Big) \\
&\qquad \le \mathbb{P}\Big(\{j' \in \hat{\mathcal{S}}\} \cap \{\tilde{b}^{j'} \notin (0, \theta^{j'})\} \cap \{N = n, \hat{j} = j, \hat{b} = b\}\Big) + \sum_{b \in (\mathcal{B} \cup \mathcal{B}_0) \cap (0, \theta^j)} 2\bar{\Phi}\big(\sqrt{d_2}/2\big) \\
&\qquad \le 2\bar{\Phi}(d_1) + 2\log_2(4p)\bar{\Phi}\big(\sqrt{d_2}/2\big) \le 2\log_2(4p)e^{-5r\beta^2/(18s\log_2(2p))},
\end{aligned}$$

where the last inequality follows from the choice of d_1 and d_2 in the statement of the theorem and the standard Gaussian tail bound used at the end of the proof of Lemma S2. By a union bound and the assumption (3), we have

$$\begin{aligned}
\mathbb{P}(z \notin \mathcal{C}) &\leq \mathbb{P}(N \leq z) + \mathbb{P}\biggl(N - \min_{j \in \hat{\mathcal{S}}}\Bigl\{t^j_{N,\tilde{b}^j} + \frac{d_2}{(\tilde{b}^j)^2}\Bigr\} > z\biggr) \\
&\leq \mathbb{P}(N \leq z) + \mathbb{P}(N > z + r) \\
&\quad + \sum_{n=z+1}^{z+\lfloor r \rfloor} \sum_{j=1}^{p} \sum_{b \in \mathcal{B}} \sum_{j'=1}^{p} \mathbb{P}\biggl(\{j' \in \hat{\mathcal{S}}\} \cap \Bigl\{n - t^{j'}_{n,\tilde{b}^{j'}} - \frac{d_2}{(\tilde{b}^{j'})^2} > z\Bigr\} \cap \{N = n, \hat{j} = j, \hat{b} = b\}\biggr) \\
&\leq \mathbb{P}(N \leq z) + g(r; N) + 4rp^2 \log_2^2(4p) e^{-5r\beta^2/(18s\log_2(2p))} \leq \alpha,
\end{aligned}$$

as required. ∎

Proof of Theorem 2. Fix $r \geq 1$ that satisfies the assumption (3). Denote $\ell_0 := 80r$. Then $\ell \geq \ell_0$. Since the output of Algorithm 1 remains unchanged if we replace $(X_t^j : t \in \mathbb{N})$ by $(-X_t^j : t \in \mathbb{N})$ for any fixed j, we may assume without loss of generality that $\theta^1 \geq \theta^2 \geq \vartheta/\sqrt{s\log_2(2p)}$. For $j \in \{1, 2\}$, we denote $b^j := \max\{b \in \mathcal{B} \cup \mathcal{B}_0 : b \leq \theta^j\}$. Since $\vartheta \geq \beta$ and $s \leq 2^{\lfloor \log_2(p) \rfloor}$, we have $b^1 \geq b^2 \geq \beta/\sqrt{s\log_2(2p)} =: b_*$. Denote

$$u := \frac{\ell_0 \beta^2}{80s\log_2(2p)} = rb_*^2 \quad \text{and} \quad \delta := \frac{a}{2\sqrt{r+\ell}}.$$

Now define the following events:

$$
\begin{aligned}
\Omega_0 &:= \{z < N \le z + r\} \\
\Omega_1 &:= \big\{t^j_{N,b} \le N - z + ub^{-2} \text{ for all } j \in [p] \text{ and } b \in \mathcal{B} \cup \mathcal{B}_0\big\}, \\
\Omega_2 &:= \Bigg\{\Bigg|A^{j',j}_{N,b} + \sum_{i=N+1}^{N+\ell} X^{j'}_i\Bigg| < a\sqrt{t^j_{N,b} + \ell} \text{ for all } b \in \mathcal{B} \cup \mathcal{B}_0, j \in [p] \\
&\qquad\qquad\qquad \text{and all } j' \in [p] \setminus \{j\} \text{ with } \big|\theta^{j'}\big| \le \delta\Bigg\}, \\
\Omega_3 &:= \big\{t^{\hat{j}}_{N,\hat{b}} \le N - z + \ell/20\big\}.
\end{aligned}
$$

Finally, we denote event

$$\Omega_4 := \Omega_{4,1} \cup \Omega_{4,2},$$

with

$$
\begin{aligned}
\Omega_{4,1} &:= \big\{\hat{j} \ne 1, 1 \in \hat{\mathcal{S}}, \tilde{b}^1 \ge b^1/\sqrt{2}\big\} \\
\Omega_{4,2} &:= \big\{\hat{j} = 1, 2 \in \hat{\mathcal{S}}, \tilde{b}^2 \ge b^2/\sqrt{2}\big\}.
\end{aligned}
$$

We note that for $j \in \{1, 2\}$, on $\Omega_{4,j}$, we have $\tilde{b}^j \in \mathcal{B} \cup \mathcal{B}_0$. Then, on the event $\bigcap_{k=0}^4 \Omega_k$, we have

$$L = \min_{j \in \hat{\mathcal{S}}}\bigg\{t^j_{N,\tilde{b}^j} + \frac{d_2}{(\tilde{b}^j)^2}\bigg\} \wedge N \le N - z + \frac{2(u + d_2)}{(b^2)^2} \le 3r + \frac{2d_2}{b_*^2} \le 8r.$$

Thus, it suffices to control the probability of $\cup_{k=0}^4 \Omega_k^{\mathrm{c}}$. First, we note that

$$\mathbb{P}(\Omega_0^{\mathrm{c}}) \le g(r; N) + \mathbb{P}(N \le z). \tag{S3}$$

On Ω_0, we have for any $j \in [p]$ and $b \in \mathcal{B} \cup \mathcal{B}_0$ that

$$\begin{aligned} t^j_{N,b} &= \operatorname*{sargmax}_{0 \le h \le N} \sum_{i=N-h+1}^{N} b(X^j_i - b/2) \le \operatorname*{sargmax}_{N-z \le h \le N} \sum_{i=N-h+1}^{N} b(X^j_i - b/2) \\ &= N - z + \operatorname*{sargmax}_{0 \le h \le z} \sum_{i=z-h+1}^{z} b(X^j_i - b/2). \end{aligned}$$

Thus, by Lemma S2 (taking $\mu = -b/2$) and a union bound, we have

$$\mathbb{P}(\Omega_0 \cap \Omega_1^{\mathrm{c}}) \le 2p \log_2(4p) e^{-u/8} = 2p \log_2(4p) e^{-rb_*^2/8}. \tag{S4}$$

Now observe that, for all $z < n \le z + r, b \in \mathcal{B} \cup \mathcal{B}_0, j \in [p]$ and $j' \in [p] \setminus \{j\}$, we have

$$A^{j',j}_{n,b} + \sum_{i=n+1}^{n+\ell} X^{j'}_i \;\Big|\; t^j_{n.b} \sim \mathcal{N}\Big(\theta^{j'}\big\{\ell + \min\big(t^j_{n,b}, n - z\big)\big\}, t^j_{n,b} + \ell\Big). \tag{S5}$$

Hence, when $\left|\theta^{j'}\right| \le \delta$, we have that

$$\mathbb{P}\left(\left|A^{j',j}_{n,b} + \sum_{i=n+1}^{n+\ell} X^{j'}_i\right| \ge a\sqrt{t^j_{n,b} + \ell} \;\middle|\; t^j_{n.b}\right) \le \mathbb{P}(|Y_1| \ge a) \le 2\mathbb{P}(Y_1 \ge a) \le e^{-a^2/8}, \tag{S6}$$

where $Y_1 \sim \mathcal{N}(\delta\sqrt{n - z + \ell}, 1)$, and where the last inequality follows from the relation $a = 2\delta\sqrt{r + \ell}$. Thus, by a union bound, we have

$$\mathbb{P}(\Omega_0 \cap \Omega_2^{\mathrm{c}}) \le 2rp^2 \log_2(4p) e^{-a^2/8}. \tag{S7}$$

Recall that $u = \ell_0 b_*^2/80$. We therefore have for any $z < n \le z + r, j \in [p]$ and $b \in \mathcal{B}$ that

$$\begin{aligned}
&\mathbb{P}\Big(\{N = n\} \cap \Omega_1 \cap \Omega_2 \cap \big\{Q_{n,b}^j \ge Q_{n,b_*}^j\big\} \cap \big\{t_{n,b}^j > n - z + \ell/20\big\} \;\Big|\; X_1^j, X_2^j, \ldots\Big) \\
&\le \mathbb{P}\Bigg(\bigcup_{\substack{j' \in [p]\setminus\{j\}: \\ |\theta^{j'}| > \delta}} \big\{|E_{n,b}^{j',j}| \ge |E_{n,b_*}^{j',j}|\big\} \cap \{N = n\} \cap \Omega_1 \cap \Omega_2 \cap \big\{t_{n,b}^j > n - z + \ell/20\big\} \;\Bigg|\; X_1^j, X_2^j, \ldots\Bigg) \\
&\le \sum_{\substack{j' \in [p]\setminus\{j\}: \\ |\theta^{j'}| > \delta}} \mathbb{P}\Big(\big\{|E_{n,b}^{j',j}| \ge |E_{n,b_*}^{j',j}|\big\} \cap \big\{n - z < t_{n,b_*}^j \le n - z + \ell/80\big\} \\
&\qquad\qquad\qquad\qquad \cap \big\{t_{n,b}^j > n - z + \ell/20\big\} \;\Big|\; X_1^j, X_2^j, \ldots\Big) \\
&\quad + \sum_{\substack{j' \in [p]\setminus\{j\}: \\ |\theta^{j'}| > \delta}} \mathbb{P}\Big(\big\{|E_{n,b}^{j',j}| \ge |E_{n,b_*}^{j',j}|\big\} \cap \big\{t_{n,b_*}^j \le n - z\big\} \cap \big\{t_{n,b}^j > n - z + \ell/20\big\} \;\Big|\; X_1^j, X_2^j, \ldots\Big) \\
&\le p \exp\Big(-\frac{\ell\delta^2}{960}\Big) = p \exp\Big(-\frac{\ell a^2}{3840(r + \ell)}\Big),
\end{aligned} \tag{S8}$$

where the final inequality follows from Lemma S4(a), applied with $U = \sum_{i=n-t_{n,b_*}^j+1}^{z} X_i^{j'}$, $V = \sum_{i=n-t_{n,b}^j+1}^{n-t_{n,b_*}^j} X_i^{j'}$, $Y = \sum_{i=z+1}^{n+\ell} X_i^{j'}$, $\alpha = \theta^{j'}$, $\phi_1 = z - n + t_{n,b_*}^j$, $\phi_2 = z - n + t_{n,b}^j$, $\phi_3 = n - z + \ell$ and $\kappa = \ell/80$, as well as Lemma S4(b), with $U = \sum_{i=n-t_{n,b}^j+1}^{z} X_i^{j'}$, $Y = \sum_{i=n-t_{n,b_*}^j+1}^{n+\ell} X_i^{j'}$, $Z = \sum_{i=z+1}^{n-t_{n,b_*}^j} X_i^{j'}$, $\alpha = \theta^{j'}$, $\phi_1 = z-n+t_{n,b}^j$, $\phi_3 = \ell + t_{n,b_*}^j$, $\phi_4 = n-z-t_{n,b_*}^j$ and $\kappa = \ell/80$. Observe that $Q_{n,\hat{b}}^{\hat{j}} \ge Q_{n,b_*}^{\hat{j}}$. Thus, by a union bound, we have

$$\begin{aligned}
&\mathbb{P}(\Omega_0 \cap \Omega_1 \cap \Omega_2 \cap \Omega_3^c) \\
&\qquad \le \sum_{j=1}^{p} \sum_{b \in \mathcal{B}} \sum_{n=z+1}^{z+\lfloor r \rfloor} \mathbb{P}\Big(\{N = n\} \cap \Omega_1 \cap \Omega_2 \cap \big\{Q_{n,b}^j \ge Q_{n,b_*}^j\big\} \cap \big\{t_{n,b}^j > n - z + \ell/20\big\}\Big) \\
&\qquad \le 2rp^2 \log_2(2p) \exp\Big(-\frac{\ell a^2}{3840(r + \ell)}\Big) \le 2rp^2 \log_2(2p) e^{-a^2/3888},
\end{aligned} \tag{S9}$$

where the last inequality follows from $r = \ell_0/80 \le \ell/80$. Recall that $d_1^2 = 5rb_*^2/9 \le$

$(b^1)^2\ell/144$. Thus, on $\Omega_0 \cap \Omega_3$, we have

$$t^{\hat{j}}_{N,\hat{b}} \leq N - z + \ell/20 \leq r + \ell/20 \leq \ell/16.$$

Hence, for any $z < n \leq z + r$, $j \in [p] \setminus \{1\}$ and $b \in \mathcal{B}$, we have

$$\begin{aligned}\mathbb{P}\bigl(&\Omega_3 \cap \{N = n, \hat{j} = j, \hat{b} = b\} \cap \Omega_{4,1}^{\mathrm{c}} \mid X_1^j, X_2^j, \ldots\bigr) \\ &\leq \mathbb{P}\biggl(\{t^j_{n,b} \leq \ell/16\} \cap \Bigl\{E^{1,j}_{n,b} - b^1\sqrt{\bigl(t^j_{n,b} + \ell\bigr)/2} < d_1\Bigr\} \biggm| X_1^j, X_2^j, \ldots\biggr) \leq \frac{1}{2}e^{-d_1^2/2}.\end{aligned} \tag{S10}$$

Here, in the final bound, we have used the facts that $E^{1,j}_{n,b} \mid t^j_{n,b} \sim \mathcal{N}\bigl(\theta^1 \min\{(n + \ell - z)(t^j_{n,b} + \ell)^{-1/2}, (t^j_{n,b} + \ell)^{1/2}\}, 1\bigr)$ and that

$$\begin{aligned}\theta^1 \min\bigl\{(n + \ell - z)(t^j_{n,b} + \ell)^{-1/2}, (t^j_{n,b} + \ell)^{1/2}\bigr\} - b^1\sqrt{\bigl(t^j_{n,b} + \ell\bigr)/2} \\ \geq \frac{4\theta^1\sqrt{\ell}}{\sqrt{17}} - \frac{b^1\sqrt{17\ell}}{4\sqrt{2}} \geq \frac{b^1\sqrt{\ell}}{6} \geq 2d_1,\end{aligned}$$

when $t^j_{n,b} \leq \ell/16$, as well as the standard Gaussian tail bound used at the end of the proof of Lemma S2. By a similar argument, we also have for any $z < n \leq z + r$ and $b \in \mathcal{B}$ that

$$\begin{aligned}\mathbb{P}\bigl(&\Omega_3 \cap \{N = n, \hat{j} = 1, \hat{b} = b\} \cap \Omega_{4,2}^{\mathrm{c}} \mid X_1^1, X_2^1, \ldots\bigr) \\ &\leq \mathbb{P}\biggl(\{t^1_{n,b} \leq \ell/16\} \cap \Bigl\{E^{2,1}_{n,b} - b^2\sqrt{\bigl(t^1_{n,b} + \ell\bigr)/2} < d_1\Bigr\} \biggm| X_1^1, X_2^1, \ldots\biggr) \leq \frac{1}{2}e^{-d_1^2/2}.\end{aligned} \tag{S11}$$

Thus, by a union bound, we have

$$\begin{aligned}\mathbb{P}(\Omega_0 \cap \Omega_3 \cap \Omega_4^{\mathrm{c}}) &= \mathbb{P}(\Omega_0 \cap \Omega_3 \cap \Omega_{4,1}^{\mathrm{c}} \cap \Omega_{4,2}^{\mathrm{c}}) \\ &\leq \sum_{j=2}^{p} \sum_{b \in \mathcal{B}} \sum_{n=z+1}^{z+\lfloor r \rfloor} \mathbb{P}\big(\Omega_3 \cap \{N = n, \hat{j} = j, \hat{b} = b\} \cap \Omega_{4,1}^{\mathrm{c}}\big) \\ &\qquad\qquad + \sum_{b \in \mathcal{B}} \sum_{n=z+1}^{z+\lfloor r \rfloor} \mathbb{P}\big(\Omega_3 \cap \{N = n, \hat{j} = 1, \hat{b} = b\} \cap \Omega_{4,2}^{\mathrm{c}}\big) \\ &\leq rp \log_2(2p) e^{-5rb_*^2/18}. \end{aligned} \tag{S12}$$

Hence combining (S3), (S4), (S7), (S9) and (S12), we conclude that

$$\mathbb{P}(L > 8r) \leq g(r; N) + \mathbb{P}(N \leq z) + 4rp^2 \log_2(4p) e^{-a^2/3888} + 3rp \log_2(4p) e^{-rb_*^2/8} \leq \alpha,$$

where the last inequality follows from the choice of a with a sufficiently large universal constant C and the assumption (3). □

Proof of Theorem 3. Fix $r \geq 1$ that satisfies the assumption (3).

(a) For $j' \in \mathcal{S}_\beta^{\mathrm{c}}$, we have $|\theta^{j'}| < b_{\min}$, so the event $\{|\tilde{b}^{j'}| \leq |\theta^{j'}|\}$ is empty. Thus by (S1), we have, for $n > z, j \in [p]$, $b \in \mathcal{B}$ and $j' \in \mathcal{S}_\beta^{\mathrm{c}}$, that

$$\mathbb{P}\big(\{j' \in \hat{\mathcal{S}}\} \cap \{N = n, \hat{j} = j, \hat{b} = b\}\big) \leq 2\bar{\Phi}(d_1).$$

Hence, by a union bound, we have

$$\begin{aligned}\mathbb{P}(\hat{\mathcal{S}} \not\subseteq \mathcal{S}_\beta) &\leq \mathbb{P}(N \leq z) + \mathbb{P}(N > z + r) \\ &\quad + \sum_{n=z+1}^{z+\lfloor r \rfloor} \sum_{j=1}^{p} \sum_{b \in \mathcal{B}} \sum_{j' \in \mathcal{S}_\beta^{\mathrm{c}}} \mathbb{P}\bigl(\{j' \in \hat{\mathcal{S}}\} \cap \{N = n, \hat{j} = j, \hat{b} = b\}\bigr) \\ &\leq \mathbb{P}(N \leq z) + g(r; N) + 4rp^2 \log_2(2p) \bar{\Phi}(d_1) \leq \alpha,\end{aligned}$$

as required.

(b) We use the events $\Omega_0, \Omega_1, \Omega_2, \Omega_3$ defined in the proof of Theorem 2. Recall from the argument immediately below (S9) that we have $t_{N,\hat{b}}^{\hat{j}} \leq \ell/16$ and $d_1 \leq \min_{j' \in \mathcal{S}} |\theta^{j'}| \sqrt{\ell}/12$ on $\Omega_0 \cap \Omega_3$. Recall also the definition of $E_{n,b}^{j',j}$ from Algorithm 1. Then, for any $z < n \leq z + r$, $j \in [p]$, $j' \in \mathcal{S} \setminus \{j\}$ and $b \in \mathcal{B}$, we have

$$\begin{aligned}&\mathbb{P}\bigl(\Omega_3 \cap \{N = n, \hat{j} = j, \hat{b} = b, j' \notin \hat{\mathcal{S}}\} \bigm| X_1^j, X_2^j, \ldots\bigr) \\ &= \mathbb{P}\Bigl(\Omega_3 \cap \{N = n, \hat{j} = j, \hat{b} = b\} \cap \bigl\{|E_{n,b}^{j',j}| < b_{\min}\sqrt{t_{n,b}^j + \ell} + d_1\bigr\} \mid X_1^j, X_2^j, \ldots\Bigr) \\ &\leq \mathbb{P}\biggl(\{t_{n,b}^j \leq \ell/16\} \cap \Bigl\{|E_{n,b}^{j',j}| - b_{\min}\sqrt{t_{n,b}^j + \ell} < d_1\Bigr\} \biggm| X_1^j, X_2^j, \ldots\biggr) \leq \frac{1}{2} e^{-d_1^2/2}, \qquad \text{(S13)}\end{aligned}$$

where, in the final bound, we have used the facts that $E_{n,b}^{j',j} \mid t_{n,b}^j \sim \mathcal{N}\bigl(\theta^{j'} \min\{(n + \ell - z)(t_{n,b}^j + \ell)^{-1/2}, (t_{n,b}^j + \ell)^{1/2}\}, 1\bigr)$ and that

$$\begin{aligned}&|\theta^{j'}| \min\bigl\{(n + \ell - z)(t_{n,b}^j + \ell)^{-1/2}, (t_{n,b}^j + \ell)^{1/2}\bigr\} - b_{\min}\sqrt{t_{n,b}^j + \ell} \\ &\qquad \geq \frac{4|\theta^{j'}|\sqrt{\ell}}{\sqrt{17}} - \frac{b_{\min}\sqrt{17\ell}}{4\sqrt{2}} \geq \frac{|\theta^{j'}|\sqrt{\ell}}{6} \geq 2d_1,\end{aligned}$$

when $t^j_{n,b} \leq \ell/16$. Hence

$$
\begin{aligned}
&\mathbb{P}(\hat{\mathcal{S}} \cup \{\hat{j}\} \not\supseteq \mathcal{S}) \\
&\quad \leq \mathbb{P}(\Omega_0^{\mathrm{c}}) + \mathbb{P}(\Omega_0 \cap \Omega_1^{\mathrm{c}}) + \mathbb{P}(\Omega_0 \cap \Omega_2^{\mathrm{c}}) + \mathbb{P}(\Omega_0 \cap \Omega_1 \cap \Omega_2 \cap \Omega_3^{\mathrm{c}}) \\
&\qquad + \sum_{n=z+1}^{z+\lfloor r \rfloor} \sum_{j=1}^{p} \sum_{b \in \mathcal{B}} \sum_{j' \in \mathcal{S} \setminus \{j\}} \mathbb{P}\big(\Omega_3 \cap \{N = n, \hat{j} = j, \hat{b} = b, j' \notin \hat{\mathcal{S}}\}\big) \\
&\quad \leq g(r; N) + \mathbb{P}(N \leq z) + 4rp^2 \log_2(4p) e^{-a^2/3888} + 3rp^2 \log_2(4p) e^{-r\beta^2/(8s\log_2(2p))} \leq \alpha,
\end{aligned}
$$

where the penultimate inequality follows from (S3), (S4), (S7), (S9) and (S13), and the last inequality follows from the choice of a with a sufficiently large universal constant C and the assumption (3). □

Proof of Proposition 4. Fix $N \in \mathcal{T}_{r,m}$ and $\psi \in \mathcal{J}_N$. We denote by $\mathbb{P}^{(n_0)}_{z,\theta}$ the restriction of $\mathbb{P}_{z,\theta}$ to the filtration $\mathcal{F}_{n_0} := \sigma(X_1, \ldots, X_{n_0})$. Denote

$$
\tilde{\Theta} := \big\{\theta \in \mathbb{R}^p : \theta^j \in \{0, 1/(8\sqrt{r})\}, |\mathrm{supp}(\theta)| = m\big\},
$$

and let $\tilde{\Theta}_{\mathrm{pa}} \subseteq \tilde{\Theta}$ be an $(m/4)$-packing set with respect to the symmetric difference metric defined above, i.e. for any $\theta, \tilde{\theta} \in \tilde{\Theta}_{\mathrm{pa}}$, we have $d\big(\mathrm{supp}(\theta), \mathrm{supp}(\tilde{\theta})\big) > m/4$. We also have $\mathrm{KL}\big(\mathbb{P}^{(z+r)}_{z,\theta}, P^{(z+r)}_{z,\tilde{\theta}}\big) = r\|\theta - \tilde{\theta}\|_2^2/2 \leq m/64$.

Enumerate $\tilde{\Theta}_{\mathrm{pa}} = \big\{\theta_{(1)}, \theta_{(2)}, \ldots, \theta_{(|\tilde{\Theta}_{\mathrm{pa}}|)}\big\}$. Let $\phi^* := \mathrm{sargmin}_{k \in [|\tilde{\Theta}_{\mathrm{pa}}|]}\, d\big(\psi, \mathrm{supp}(\theta_{(k)})\big)$.

Note that ϕ^* is also $\mathcal{F}_N$-measurable. Then for any $z \in \mathbb{N}_0$,

$$
\begin{aligned}
\sup_{\theta\in\Theta_{r,m}} \mathbb{E}_{z,\theta} d\big(\psi, \mathrm{supp}(\theta)\big) &\geq \frac{m}{8|\tilde{\Theta}_{\mathrm{pa}}|} \sum_{k=1}^{|\tilde{\Theta}_{\mathrm{pa}}|} \mathbb{P}_{z,\theta_{(k)}}\Big(d\big(\psi, \mathrm{supp}(\theta_{(k)})\big) > \frac{m}{8}\Big) \\
&\geq \frac{m}{8|\tilde{\Theta}_{\mathrm{pa}}|} \sum_{k=1}^{|\tilde{\Theta}_{\mathrm{pa}}|} \mathbb{P}_{z,\theta_{(k)}}(\phi^* \neq k) \\
&= \frac{m}{8}\bigg\{1 - \frac{1}{|\tilde{\Theta}_{\mathrm{pa}}|} \sum_{k=1}^{|\tilde{\Theta}_{\mathrm{pa}}|} \mathbb{P}_{z,\theta_{(k)}}(\phi^* = k)\bigg\} \\
&\geq \frac{m}{8}\bigg\{\frac{3}{4} - \frac{1}{|\tilde{\Theta}_{\mathrm{pa}}|} \sum_{k=1}^{|\tilde{\Theta}_{\mathrm{pa}}|} \mathbb{P}_{z,\theta_{(k)}}(\phi^* = k, N \leq z+r)\bigg\} \\
&= \frac{m}{8}\bigg\{\frac{3}{4} - \frac{1}{|\tilde{\Theta}_{\mathrm{pa}}|} \sum_{k=1}^{|\tilde{\Theta}_{\mathrm{pa}}|} \mathbb{P}^{(z+r)}_{z,\theta_{(k)}}(\phi^* = k, N \leq z+r)\bigg\}. \qquad \text{(S14)}
\end{aligned}
$$

Now set

$$
\tilde{\phi}^* := \begin{cases} \phi^* & \text{if } N \leq z+r \\ 1 & \text{if } N > z+r. \end{cases}
$$

Then $\tilde{\phi}^*$ is $\mathcal{F}_{z+r}$-measurable and by Fano's inequality (Yu 1997, Lemma 3), we have

$$
\begin{aligned}
\frac{1}{|\tilde{\Theta}_{\mathrm{pa}}|} \sum_{k=1}^{|\tilde{\Theta}_{\mathrm{pa}}|} \mathbb{P}^{(z+r)}_{z,\theta_{(k)}}(\phi^* = k, N \leq z+r) &\leq \frac{1}{|\tilde{\Theta}_{\mathrm{pa}}|} \sum_{k=1}^{|\tilde{\Theta}_{\mathrm{pa}}|} \mathbb{P}^{(z+r)}_{z,\theta_{(k)}}(\tilde{\phi}^* = k) \\
&\leq \frac{\log 2 + |\tilde{\Theta}_{\mathrm{pa}}|^{-2} \sum_{j,k=1}^{|\tilde{\Theta}_{\mathrm{pa}}|} \mathrm{KL}\big(\mathbb{P}^{(z+r)}_{z,\theta_{(j)}}, P^{(z+r)}_{z,\theta_{(k)}}\big)}{\log |\tilde{\Theta}_{\mathrm{pa}}|} \\
&\leq \frac{\log 2 + m/64}{\log |\tilde{\Theta}_{\mathrm{pa}}|}. \qquad \text{(S15)}
\end{aligned}
$$

By Massart (2007, Lemma 4.7), there exists an $(m/4)$-packing set with

$$\log|\tilde{\Theta}_{\mathrm{pa}}| \geq m/8. \tag{S16}$$

Combining (S14), (S15) and (S16), we conclude that

$$\begin{aligned}\sup_{z\in\mathbb{N}_0,\theta\in\Theta_{r,m}} \mathbb{E}_{z,\theta}\, d\big(\psi,\mathrm{supp}(\theta)\big) &\geq \frac{m}{8}\biggl(\frac{3}{4}-\frac{\log 2+m/64}{m/8}\biggr)\\ &\geq \frac{m}{8}\biggl(\frac{3}{4}-\frac{8\log 2}{m}-\frac{1}{8}\biggr)\geq \frac{m}{32},\end{aligned}$$

where we have used the assumption that $m \geq 15$ in the final inequality. □

Proof of Proposition 5. Following the proof of Chen et al. (2022, Theorem 1(b)) up to, but not including, (16), we have for every $j \in [p]$, $b \in \mathcal{B} \cup \mathcal{B}_0$ that

$$\mathbb{P}\Bigl(\max_{1\leq n\leq z}(bA^{j,j}_{n,b}-b^2 t^j_{n,b}/2)\geq T^{\mathrm{diag}}\Bigr)\leq 1-\bigl(1-e^{-T^{\mathrm{diag}}}\bigr)^z\leq ze^{-T^{\mathrm{diag}}}. \tag{S17}$$

It follows by a union bound that

$$\begin{aligned}\mathbb{P}\Bigl(\max_{1\leq n\leq z} S^{\mathrm{diag}}_n \geq T^{\mathrm{diag}}\Bigr) &= \mathbb{P}\Bigl(\max_{1\leq n\leq z}\max_{j\in[p]}\max_{b\in\mathcal{B}\cup\mathcal{B}_0}(bA^{j,j}_{n,b}-b^2t^j_{n,b}/2)\geq T^{\mathrm{diag}}\Bigr)\\ &\leq zp|\mathcal{B}\cup\mathcal{B}_0|e^{-T^{\mathrm{diag}}}\leq \frac{\alpha}{4}.\end{aligned} \tag{S18}$$

Next, for every $j \in [p], j' \in [p]\setminus\{j\}, b \in \mathcal{B}$ and $n \in [z]$, we have $\Lambda^{j',j}_{n,b} \mid \tau \sim \mathcal{N}(0,\tau^j_{n,b})$, so

$(\Xi_{n,b}^{j',j})^2 \mid \tau_{n,b}^j \leq_{\mathrm{st}} \chi_1^2$. Thus, by another union bound, we have

$$\begin{aligned}\mathbb{P}\Big(\max_{n\in[z]} S_n^{\mathrm{off}} \geq T^{\mathrm{off}}\Big) &= \mathbb{P}\Bigg(\max_{n\in[z]}\max_{j\in[p]}\max_{b\in\mathcal{B}} \sum_{j'\in[p]\setminus\{j\}} (\Xi_{n,b}^{j',j})^2 \mathbb{1}_{\{|\Xi_{n,b}^{j',j}|\geq\tilde{a}\}} \geq T^{\mathrm{off}}\Bigg)\\ &\leq \mathbb{P}\Big(\max_{n\in[z]}\max_{j\in[p]}\max_{b\in\mathcal{B}}\max_{j'\in[p]\setminus\{j\}} |\Xi_{n,b}^{j',j}| \geq \tilde{a}\Big)\\ &\leq zp^2|\mathcal{B}|e^{-\tilde{a}^2/2} \leq \frac{\alpha}{4}. \end{aligned} \tag{S19}$$

From (S18) and (S19), we deduce that

$$\mathbb{P}(N \leq z) \leq \alpha/2. \tag{S20}$$

On the other hand, for a sufficiently large universal constant $C' > 0$, we have

$$\begin{aligned} r_1 &= \frac{C's\log_2(2p)\log\{p\gamma\alpha^{-1}(\beta^{-2}\vee 1)\}}{\beta^2} + 2\\ &\geq \left\{\frac{24T^{\mathrm{off}}\log_2(2p)}{\vartheta^2} \vee \frac{12\tilde{a}^2 s\log_2(2p)}{\vartheta^2} \vee \frac{24T^{\mathrm{diag}} s\log_2(2p)}{\beta^2}\right\} + 2. \end{aligned}$$

Thus, by Proposition S3 and by increasing the value of C if necessary, we have for every $r \geq r_1$ that

$$\begin{aligned} g(r;N) + 4rp^2\log_2^2(4p)e^{-r\beta^2/(8s\log_2(2p))} &\leq 5rp^2\log_2^2(4p)\exp\left\{-\frac{r\beta^2}{48s\log_2(2p)}\right\}\\ &\leq \frac{240sp^2\log_2^3(4p)}{\beta^2}\frac{r\beta^2}{48s\log_2(2p)}\exp\left\{-\frac{r\beta^2}{48s\log_2(2p)}\right\}\\ &\leq \frac{240sp^2\log_2^3(4p)}{\beta^2}\exp\left\{-\frac{r\beta^2}{96s\log_2(2p)}\right\} \leq \frac{\alpha}{4}, \end{aligned} \tag{S21}$$

where the penultimate inequality follows from the fact that $xe^{-x} \leq e^{-x/2}$ for $x \geq 0$. The desired result follows by combining (S20) and (S21). $\square$

S2. AUXILIARY RESULTS

Proposition S1. *Let $X_1, X_2, \ldots$ be independent random variables with $X_1, \ldots, X_z \overset{\text{iid}}{\sim} \mathcal{N}(0,1)$ and $X_{z+1}, X_{z+2}, \ldots \overset{\text{iid}}{\sim} \mathcal{N}(\theta, 1)$. Assume that $0 < b \leq \theta$ and let $t_{n,b}$ be defined as in (1) in the main text for $n \in \mathbb{N}$. Then for any $\alpha \in (0,1)$, and any stopping time N satisfying $\mathbb{P}(N < z) \leq \alpha/2$, we have that the confidence interval*

$$\mathcal{C}_0 := \left[N - t_{N,b} - \frac{4\{\Phi^{-1}(1-\alpha/4)\}^2}{b^2},\, N\right]$$

satisfies $\mathbb{P}(z \in \mathcal{C}_0) \geq 1 - \alpha$.

Remark. *We could also replace $4\{\Phi^{-1}(1-\alpha/4)\}^2/b^2$ by $8\log(2/\alpha)/b^2$ in the confidence interval construction, if we apply the final bound from Lemma S2 in (S22) of the proof below.*

Proof. For $n \in \mathbb{N}$, define $R_{n,b} := \max\{R_{n-1,b} + b(X_n - b/2), 0\}$, with $R_{0,b} = 0$. By Chen et al. (2022, Lemma 2 in the supplement), we have $t_{N,b} = \min\{i : 0 \leq i \leq N, R_{N-i,b} = 0\} = \mathrm{sargmax}_{0 \leq h \leq N} \sum_{i=N-h+1}^{N} b(X_i - b/2)$. Let $U_{n,b} := \sum_{i=z+1}^{z+n}(X_i - b/2)$ for $n \in \mathbb{N}$, with $U_{0,b} := 0$. Then $R_{n+z,b} \geq bU_{n,b}$ for all $n \in \mathbb{N}$. Hence, for $y \in [0, \infty)$, we have

$$\mathbb{P}(N - t_{N,b} - y \geq z) \leq \mathbb{P}\Big(\inf_{n \in \mathbb{N}_0 : n \geq z+y} R_{n,b} = 0\Big) \leq \mathbb{P}\Big(\inf_{n \in \mathbb{N}_0 : n \geq y} U_{n,b} \leq 0\Big) \leq 2\bar{\Phi}\Big(\sqrt{y}(\theta - b/2)\Big), \tag{S22}$$

where the last inequality follows from Lemma S2. Thus, if we choose $y = 4\{\Phi^{-1}(1 - \alpha/4)\}^2/b^2$, then we are guaranteed that $\mathbb{P}(N - t_{N,b} - y > z) \leq \alpha/2$. Combining this with

the assumption that $\mathbb{P}(N < z) \leq \alpha/2$, the desired result follows. $\square$

Lemma S2. *Let* $Y_1, Y_2, \ldots \overset{\text{iid}}{\sim} \mathcal{N}(\mu, 1)$*. Define* $U_n := \sum_{i=1}^n Y_i$ *for* $n \in \mathbb{N}_0$*, and let* $\xi := \operatorname{sargmin}_{n\in\mathbb{N}_0} \mu U_n$*. Then, for* $y \in [0, \infty)$*, we have*

$$\mathbb{P}(\xi \geq y) \leq \mathbb{P}\Bigl(\inf_{n\in\mathbb{N}_0: n\geq y} \mu U_n \leq 0\Bigr) \leq 2\bar{\Phi}\bigl(\sqrt{y}|\mu|\bigr) \leq e^{-y\mu^2/2}.$$

Proof. The first inequality holds since $\mu U_\xi \leq \mu U_0 = 0$. For the second and third inequalities, we may assume without loss of generality that $\mu > 0$, since the result is clear when $\mu = 0$, and if $\mu < 0$ then the result will follow from the corresponding result with $\mu > 0$ by setting $Y_i' := -Y_i$ for $i \in \mathbb{N}$. Note that $(U_n - n\mu)_{n\in\mathbb{N}_0}$ is a standard Gaussian random walk starting at 0. Let $(B_t)_{t\in[0,\infty)}$ denote a standard Brownian motion starting at 0. Then, we have for any $y \in \mathbb{N}_0$ and $u > 0$ that

$$\mathbb{P}\biggl(\inf_{n\in\mathbb{N}_0: n\geq y} U_n \leq 0 \biggm| U_y = u\biggr) \leq \mathbb{P}\biggl\{\inf_{t\in[y,\infty)} (B_t + t\mu) \leq 0 \biggm| B_y = u\biggr\} \leq e^{-2u\mu}, \tag{S23}$$

where the final inequality follows from Siegmund (1986, Proposition 2.4 and Equation (2.5)). Thus, for $y \in [0, \infty)$, we have

$$\begin{aligned}
\mathbb{P}\biggl(\inf_{n\in\mathbb{N}_0: n\geq y} U_n \leq 0\biggr) &= \mathbb{P}\bigl(U_{\lceil y\rceil} \leq 0\bigr) + \mathbb{E}\biggl\{\mathbb{P}\biggl(\inf_{n\in\mathbb{N}_0: n\geq \lceil y\rceil} U_n \leq 0 \biggm| U_{\lceil y\rceil}\biggr)\mathbb{1}_{\{U_{\lceil y\rceil} > 0\}}\biggr\} \\
&\leq \bar{\Phi}\Bigl(\sqrt{\lceil y\rceil}\mu\Bigr) + \int_0^\infty \frac{1}{\sqrt{2\pi\lceil y\rceil}} e^{-\frac{(u-\lceil y\rceil\mu)^2}{2\lceil y\rceil}} e^{-2u\mu}\, du \\
&= 2\bar{\Phi}\Bigl(\sqrt{\lceil y\rceil}\mu\Bigr) \leq 2\bar{\Phi}\bigl(\sqrt{y}\mu\bigr) \leq e^{-y\mu^2/2},
\end{aligned}$$

where the first inequality follows from (S23) and the fact that $U_{\lceil y\rceil} \sim \mathcal{N}(\lceil y\rceil\mu, \lceil y\rceil)$ and the last inequality follows from the standard normal distribution tail bound $\bar{\Phi}(x) \leq e^{-x^2/2}/2$ for $x \geq 0$. $\square$

In Proposition S3, we assume the Gaussian data generating mechanism given at the beginning of Section 3 of the main text, and show that for the $\texttt{ocd}'$ base procedure, the quantity $g(r;N)$ from (2) has essentially the same form as the final term in (3).

Proposition S3. *Assume that θ has an effective sparsity of $s := s(\theta) \geq 2$. Then, the output N from $\texttt{ocd}'$, with inputs $(X_t)_{t\in\mathbb{N}}$, $0 < \beta \leq \vartheta$, $\tilde{a} > 0$, $T^{\mathrm{diag}} > 0$ and $T^{\mathrm{off}} > 0$, satisfies*

$$\mathbb{P}_{z,\theta,\Sigma}\big(N > z + r\big) \leq p\exp\biggl\{-\frac{\beta^2(r-1)}{24s\log_2(2p)}\biggr\},$$

for all $r \geq \Bigl\{\frac{24T^{\mathrm{off}}\log_2(2p)}{\vartheta^2} \vee \frac{12\tilde{a}^2 s\log_2(2p)}{\vartheta^2} \vee \frac{24T^{\mathrm{diag}} s\log_2(2p)}{\beta^2}\Bigr\} + 2.$

Proof. For $\theta \in \mathbb{R}^p$ with effective sparsity $s(\theta)$, there is at most one coordinate in θ of magnitude larger than $\vartheta/\sqrt{2}$, so there exists $b_* \in \bigl\{\beta/\sqrt{s(\theta)\log_2(2p)}, -\beta/\sqrt{s(\theta)\log_2(2p)}\bigr\} \subseteq \mathcal{B}$ such that

$$\mathcal{J} := \Bigl\{j \in [p] : \theta^j/b_* \geq 1 \text{ and } |\theta^j| \leq \vartheta/\sqrt{2}\Bigr\}$$

has cardinality at least $s(\theta)/2$. Note that the condition $\theta^j/b_* \geq 1$ above ensures that $\{\theta^j : j \in \mathcal{J}\}$ all have the same sign as b_*. By Chen et al. (2022, Proposition 8), we have on the event $\{N > z\}$ that

$$q(X_1,\ldots,X_z,\theta) := \max\bigl\{t^j_{z,b_*} : j \in \mathcal{J}\bigr\} \leq \frac{8T^{\mathrm{diag}} s\log_2(2p)}{\beta^2}. \tag{S24}$$

We now fix

$$r \geq \biggl\{\frac{24T^{\mathrm{off}}\log_2(2p)}{\vartheta^2} \vee \frac{12\tilde{a}^2 s\log_2(2p)}{\vartheta^2} \vee \frac{24T^{\mathrm{diag}} s\log_2(2p)}{\beta^2}\biggr\} + 2 =: r_0. \tag{S25}$$

For $j \in \mathcal{J}$, define the event

$$\Omega_r^j := \{t^j_{z+\lfloor r\rfloor, b_*} > 2\lfloor r\rfloor/3\}.$$

By applying Chen et al. (2022, Lemma 2) to $t^j_{z+\lfloor r\rfloor, b_*}$, we have for $j \in \mathcal{J}$ that

$$\begin{aligned} t^j_{z+\lfloor r\rfloor, b_*} &= \operatorname*{sargmax}_{0\le h\le z+\lfloor r\rfloor} \sum_{i=z+\lfloor r\rfloor-h+1}^{z+\lfloor r\rfloor} b_*(X_i^j - b_*/2) \ge \operatorname*{sargmax}_{0\le h\le \lfloor r\rfloor} \sum_{i=z+\lfloor r\rfloor-h+1}^{z+\lfloor r\rfloor} b_*(X_i^j - b_*/2) \\ &= \operatorname*{sargmax}_{0\le h\le \lfloor r\rfloor} \sum_{i=z+1}^{z+\lfloor r\rfloor-h} -b_*(X_i^j - b_*/2) = \lfloor r\rfloor - \operatorname*{largmax}_{0\le h\le \lfloor r\rfloor} \sum_{i=z+1}^{z+h} -b_*(X_i^j - b_*/2). \end{aligned}$$

Recall that $X^j_{z+1}, X^j_{z+2}, \ldots \overset{\text{iid}}{\sim} \mathcal{N}(\theta^j, 1)$. Hence, by applying Lemma S2 with $\mu = |b_*|/2$ and $y = \lfloor r\rfloor/3$, we have for each $j \in \mathcal{J}$ that

$$\begin{aligned} \mathbb{P}\big\{(\Omega_r^j)^{\mathrm{c}}\big\} &= \mathbb{P}\left(t^j_{z+\lfloor r\rfloor, b_*} \le \frac{2\lfloor r\rfloor}{3}\right) \\ &\le \mathbb{P}\left(\operatorname*{largmax}_{0\le h\le \lfloor r\rfloor} \sum_{i=z+1}^{z+h} -b_*(X_i^j - b_*/2) \ge \frac{\lfloor r\rfloor}{3}\right) \\ &\le \mathbb{P}\left(\sup_{h\ge \lfloor r\rfloor/3} \sum_{i=z+1}^{z+h} -\mathrm{sgn}(b_*)(X_i^j - b_*/2) \ge 0\right) \le \exp\big(-b_*^2\lfloor r\rfloor/24\big). \end{aligned} \tag{S26}$$

We now work on the event Ω_r^j, for some fixed $j \in \mathcal{J}$. We note that (S25) guarantees that $r \ge 2$, and thus $t^j_{z+\lfloor r\rfloor, b_*} \ge \lceil 2\lfloor r\rfloor/3\rceil \ge 2$. Then, by (S24) and (S25), we have $r_0 > 3t^j_{z,b_*}$, and hence by Chen et al. (2022, Lemma 9),

$$\frac{\lfloor r\rfloor}{3} < \frac{t^j_{z+\lfloor r\rfloor, b_*}}{2} \le \tau^j_{z+\lfloor r\rfloor, b_*} \le \frac{3t^j_{z+\lfloor r\rfloor, b_*}}{4} \le \frac{3\big(t^j_{z,b_*} + r\big)}{4} < r.$$

We conclude that

$$2/3 \leq \lfloor r \rfloor/3 < \tau^{j}_{z+\lfloor r \rfloor, b_*} \leq \lfloor r \rfloor. \tag{S27}$$

Recall that $\Lambda^{\cdot,j}_{z+\lfloor r \rfloor, b_*} \in \mathbb{R}^p$ records the tail CUSUM statistics with tail length $\tau^{j}_{z+\lfloor r \rfloor, b_*}$. We observe by (S27) that only post-change observations are included in $\Lambda^{\cdot,j}_{z+\lfloor r \rfloor, b_*}$. Hence we have that

$$\Lambda^{j',j}_{z+\lfloor r \rfloor, b_*} \;\middle|\; \tau^{j}_{z+\lfloor r \rfloor, b_*} \overset{\text{ind}}{\sim} \mathcal{N}\big(\theta^{j'} \tau^{j}_{z+\lfloor r \rfloor, b_*}, \tau^{j}_{z+\lfloor r \rfloor, b_*}\big) \tag{S28}$$

for $j' \in [p] \setminus \{j\}$. By the definition of the effective sparsity of θ, the set

$$\mathcal{L}^j := \left\{ j' \in [p] \setminus [j] : |\theta^{j'}| \geq \frac{\vartheta}{\sqrt{s \log_2(2p)}} \right\}$$

has cardinality at least $s - 1$. Hence, by (S27), for all $j' \in \mathcal{L}^j$,

$$|\theta^{j'}| \sqrt{\tau^{j}_{z+\lfloor r \rfloor, b_*}} > \sqrt{\frac{\vartheta^2 \lfloor r \rfloor}{3s \log_2(2p)}} =: \tilde{a}_r.$$

We then observe, from (S25), that

$$\tilde{a}_r > 2\tilde{a}. \tag{S29}$$

Hence, from (S28), we have for all $j' \in \mathcal{L}^j$ that

$$\mathbb{P}\left(\Omega^j_r \cap \left\{ |\Lambda^{j',j}_{z+\lfloor r \rfloor, b_*}| < \frac{1}{2} \tilde{a}_r \sqrt{\tau^{j}_{z+\lfloor r \rfloor, b_*}} \right\} \;\middle|\; \tau^{j}_{z+\lfloor r \rfloor, b_*} \right) \leq \frac{1}{2} e^{-\tilde{a}_r^2/8}. \tag{S30}$$

We denote

$$U^j := \bigcap_{j' \in \mathcal{L}^j} \left\{ |\Lambda^{j',j}_{z+\lfloor r \rfloor, b_*}| \geq \frac{1}{2} \tilde{a}_r \sqrt{\tau^{j}_{z+\lfloor r \rfloor, b_*}} \right\}.$$

Thus, by a union bound, we have

$$\mathbb{P}\big(\Omega_r^j \cap (U^j)^{\mathrm{c}}\big) \leq \frac{p}{2} e^{-\tilde{a}_r^2/8}. \tag{S31}$$

Moreover, on the event $\Omega_r^j \cap U^j$, we have

$$\begin{aligned}\sum_{j'\in[p]:j'\neq j} \frac{\big(\Lambda^{j',j}_{z+\lfloor r\rfloor,b_*}\big)^2}{\tau^j_{z+\lfloor r\rfloor,b_*} \vee 1} \mathbb{1}_{\Big\{|\Lambda^{j',j}_{z+\lfloor r\rfloor,b_*}| \geq \tilde{a}\sqrt{\tau^j_{z+\lfloor r\rfloor,b_*}}\Big\}} &\geq \sum_{j'\in\mathcal{L}^j} \frac{\big(\Lambda^{j',j}_{z+\lfloor r\rfloor,b_*}\big)^2}{\tau^j_{z+\lfloor r\rfloor,b_*}} \\ &\geq \frac{\tilde{a}_r^2}{4}|\mathcal{L}^j| \geq \frac{\vartheta^2\lfloor r\rfloor}{24\log_2(2p)} \geq T^{\mathrm{off}}, \end{aligned} \tag{S32}$$

where the penultimate inequality uses the fact that $|\mathcal{L}^j| \geq s-1$ and the last inequality follows from (S25). We now denote

$$\tilde{E}_r^j := \Bigg\{ \sum_{j'\in[p]:j'\neq j} \frac{\big(\Lambda^{j',j}_{z+\lfloor r\rfloor,b_*}\big)^2}{\tau^j_{z+\lfloor r\rfloor,b_*} \vee 1} \mathbb{1}_{\Big\{|\Lambda^{j',j}_{z+\lfloor r\rfloor,b_*}| \geq \tilde{a}\sqrt{\tau^j_{z+\lfloor r\rfloor,b_*}}\Big\}} < T^{\mathrm{off}} \Bigg\}.$$

By (S32), we have $\Omega_r^j \cap \tilde{E}_r^j \subseteq \Omega_r^j \cap (U^j)^{\mathrm{c}}$. Thus, by (S26) and (S31) we have that

$$\begin{aligned}\mathbb{P}\big(N > z + r\big) &\leq \mathbb{P}\big(N > z + \lfloor r\rfloor\big) \leq \mathbb{P}\Bigg(\bigcap_{j\in\mathcal{J}} \tilde{E}_r^j\Bigg) \leq \min_{j\in\mathcal{J}} \mathbb{P}(\tilde{E}_r^j) \\ &\leq \min_{j\in\mathcal{J}}\Big\{\mathbb{P}\big((U^j)^{\mathrm{c}} \cap \Omega_r^j\big) + \mathbb{P}\big((\Omega_r^j)^{\mathrm{c}}\big)\Big\} \\ &\leq \frac{p}{2}\exp\bigg\{-\frac{\vartheta^2(r-1)}{24s\log_2(2p)}\bigg\} + \exp\bigg\{-\frac{\beta^2(r-1)}{24s\log_2(2p)}\bigg\} \\ &\leq p\exp\bigg\{-\frac{\beta^2(r-1)}{24s\log_2(2p)}\bigg\}. \end{aligned}$$

as desired. □

Lemma S4. *Let $U \sim \mathcal{N}(0, \phi_1)$, $V \sim \mathcal{N}(0, \phi_2 - \phi_1)$, $Y \sim \mathcal{N}(\alpha\phi_3, \phi_3)$ and $Z \sim \mathcal{N}(\alpha\phi_4, \phi_4)$*

be independent random variables.

(a) *Assume that* $\min\{\phi_2, \phi_3\}/4 \geq \kappa \geq \phi_1 \geq 0$ *for some* $\kappa > 0$. *Then*

$$\mathbb{P}\biggl(\frac{|U+V+Y|}{\sqrt{\phi_2+\phi_3}} \geq \frac{|U+Y|}{\sqrt{\phi_1+\phi_3}}\biggr) \leq \exp\biggl(-\frac{\kappa\alpha^2}{6}\biggr).$$

(b) *Assume that* $\min\{\phi_1, \phi_3\}/4 \geq \kappa \geq \phi_4 \geq 0$ *for some* $\kappa > 0$. *Then*

$$\mathbb{P}\biggl(\frac{|U+Y+Z|}{\sqrt{\phi_1+\phi_3+\phi_4}} \geq \frac{|Y|}{\sqrt{\phi_3}}\biggr) \leq \exp\biggl(-\frac{\kappa\alpha^2}{12}\biggr).$$

Proof. The case $\alpha = 0$ is trivial in both cases, so without loss of generality, we may assume $\alpha > 0$ throughout the rest of the proof.

(a) Let

$$W_1 := \bigl(\sqrt{\phi_2+\phi_3} - \sqrt{\phi_1+\phi_3}\bigr)(U+Y) - \sqrt{\phi_1+\phi_3}\,V,$$

so that

$$W_1 \sim \mathcal{N}\Bigl(\alpha\phi_3\bigl(\sqrt{\phi_2+\phi_3} - \sqrt{\phi_1+\phi_3}\bigr), \bigl\{\bigl(\sqrt{\phi_2+\phi_3} - \sqrt{\phi_1+\phi_3}\bigr)^2 + \phi_2 - \phi_1\bigr\}(\phi_1+\phi_3)\Bigr).$$

Hence, by the standard Gaussian tail bound used at the end of the proof of Lemma S2, we have

$$\mathbb{P}(W_1 \leq 0) \leq \frac{1}{2}e^{-\alpha^2/(2w_1)}, \tag{S33}$$

where $w_1 := \frac{\phi_1+\phi_3}{\phi_3^2}\bigl(1+\frac{\phi_2-\phi_1}{(\sqrt{\phi_2+\phi_3}-\sqrt{\phi_1+\phi_3})^2}\bigr)$. Then

$$\begin{aligned} w_1 &= \frac{\phi_1+\phi_3}{\phi_3^2}\left(1+\frac{\left(\sqrt{\phi_2+\phi_3}+\sqrt{\phi_1+\phi_3}\right)^2}{\phi_2-\phi_1}\right) \\ &\leq \frac{5}{16\kappa}\left(1+\frac{\left(\sqrt{8\kappa}+\sqrt{5\kappa}\right)^2}{3\kappa}\right) \leq \frac{3}{\kappa}, \end{aligned} \tag{S34}$$

where the first inequality holds because w_1 is increasing in ϕ_1 and decreasing in both ϕ_2 and ϕ_3. Hence, using the fact that $-(U+V+Y) \leq_{\mathrm{st}} U+V+Y$, as well as (S33) and (S34), we have

$$\begin{aligned} \mathbb{P}\left(\frac{|U+V+Y|}{\sqrt{\phi_2+\phi_3}} \geq \frac{|U+Y|}{\sqrt{\phi_1+\phi_3}}\right) &\leq \mathbb{P}\left(\left\{\frac{U+Y}{\sqrt{\phi_1+\phi_3}} \leq \frac{U+V+Y}{\sqrt{\phi_2+\phi_3}}\right\} \cap \{U+V+Y \geq 0\}\right) \\ &\quad + \mathbb{P}\left(\left\{\frac{U+Y}{\sqrt{\phi_1+\phi_3}} \leq -\frac{U+V+Y}{\sqrt{\phi_2+\phi_3}}\right\} \cap \{U+V+Y < 0\}\right) \\ &\leq 2\mathbb{P}\left(\frac{U+Y}{\sqrt{\phi_1+\phi_3}} \leq \frac{U+V+Y}{\sqrt{\phi_2+\phi_3}}\right) \\ &= 2\mathbb{P}(W_1 \leq 0) \leq \exp\left(-\frac{\kappa\alpha^2}{6}\right), \end{aligned}$$

as required.

(b) Let

$$W_2 := \bigl(\sqrt{\phi_1+\phi_3+\phi_4}-\sqrt{\phi_3}\bigr)Y - \sqrt{\phi_3}(U+Z),$$

so that

$$W_2 \sim \mathcal{N}\Bigl(\alpha\phi_3\sqrt{\phi_1+\phi_3+\phi_4} - \alpha(\phi_3+\phi_4)\sqrt{\phi_3}, \bigl\{\bigl(\sqrt{\phi_1+\phi_3+\phi_4}-\sqrt{\phi_3}\bigr)^2+\phi_1+\phi_4\bigr\}\phi_3\Bigr).$$

Note that the assumption guarantees that $\mathbb{E}(W_2) > 0$. Hence, by the standard Gaussian

tail bound used at the end of the proof of Lemma S2, we have

$$\mathbb{P}(W_2 \leq 0) \leq \frac{1}{2} e^{-\alpha^2/(2w_2)}, \tag{S35}$$

where

$$w_2 := \frac{(\sqrt{\phi_1+\phi_3+\phi_4} - \sqrt{\phi_3})^2 + \phi_1 + \phi_4}{\left(\sqrt{\phi_3(\phi_1+\phi_3+\phi_4)} - \phi_3 - \phi_4\right)^2}.$$

Calculating the partial derivatives of w_2 with respect to ϕ_1, ϕ_3 and ϕ_4 and simplifying the expressions, we have

$$\begin{aligned}
\frac{\partial w_2}{\partial \phi_1} &= \frac{(\phi_3+\phi_4)\sqrt{\phi_3} - (\phi_3 + 2\phi_4)\sqrt{\phi_1+\phi_3+\phi_4}}{\sqrt{\phi_1+\phi_3+\phi_4}\left(\sqrt{\phi_3(\phi_1+\phi_3+\phi_4)} - \phi_3 - \phi_4\right)^3} \leq 0, \\
\frac{\partial w_2}{\partial \phi_3} &= \frac{-\left(\sqrt{\phi_1+\phi_3+\phi_4} - \sqrt{\phi_3}\right)^2\left[3\phi_1 + \phi_4 + \left(\sqrt{\phi_1+\phi_3+\phi_4} - \sqrt{\phi_3}\right)^2\right]}{2\sqrt{\phi_3(\phi_1+\phi_3+\phi_4)}\left(\sqrt{\phi_3(\phi_1+\phi_3+\phi_4)} - \phi_3 - \phi_4\right)^3} \leq 0, \\
\frac{\partial w_2}{\partial \phi_4} &= \frac{2\phi_1\left(2\sqrt{\phi_1+\phi_3+\phi_4} - \sqrt{\phi_3}\right)^2 + 3(\phi_3+\phi_4)\left(\sqrt{\phi_1+\phi_3+\phi_4} - \sqrt{\phi_3}\right)^2}{2(\phi_1+\phi_3+\phi_4)\left(\sqrt{\phi_3(\phi_1+\phi_3+\phi_4)} - \phi_3 - \phi_4\right)^3} \\
&\quad + \frac{(\phi_1+\phi_4)(\phi_3+\phi_4)}{2(\phi_1+\phi_3+\phi_4)\left(\sqrt{\phi_3(\phi_1+\phi_3+\phi_4)} - \phi_3 - \phi_4\right)^3} \geq 0.
\end{aligned}$$

Thus w_2 is increasing in ϕ_4 and decreasing in both ϕ_1 and ϕ_3 and hence

$$w_2 \leq \frac{6}{\kappa}. \tag{S36}$$

Hence, using the fact that $-(U+Y+Z) \leq_{\mathrm{st}} U+Y+Z$, as well as (S35) and (S36), we

have

$$\begin{aligned}\mathbb{P}\biggl(\frac{|U+Y+Z|}{\sqrt{\phi_1+\phi_3+\phi_4}} \geq \frac{|Y|}{\sqrt{\phi_3}}\biggr) &\leq \mathbb{P}\biggl(\biggl\{\frac{Y}{\sqrt{\phi_3}} \leq \frac{U+Y+Z}{\sqrt{\phi_1+\phi_3+\phi_4}}\biggr\} \cap \{U+Y+Z \geq 0\}\biggr) \\ &\quad + \mathbb{P}\biggl(\biggl\{\frac{Y}{\sqrt{\phi_3}} \leq -\frac{U+Y+Z}{\sqrt{\phi_1+\phi_3+\phi_4}}\biggr\} \cap \{U+Y+Z < 0\}\biggr) \\ &\leq 2\mathbb{P}\biggl(\frac{Y}{\sqrt{\phi_3}} \leq \frac{U+Y+Z}{\sqrt{\phi_1+\phi_3+\phi_4}}\biggr) \\ &= 2\mathbb{P}(W_2 \leq 0) \leq \exp\biggl(-\frac{\kappa\alpha^2}{12}\biggr),\end{aligned}$$

as required. □

S3. THE `ocd` AND `ocd′` BASE PROCEDURES

Algorithms S1 and S2, which are taken from Chen et al. (2022), provide two options for a base online changepoint detection procedure. The `ocd` algorithm is recommended for practical use, while its variant, the `ocd′` algorithm, satisfies the key condition (3) that underpins our theoretical results in Section 3 of the main text.

In order to help make the paper self-contained, and to aid interpretability, we remark that the input parameter $\tilde{a}$ represents a thresholding level employed in the definition of the quantity Q_b^j in Algorithm S1 and $\tilde{Q}_b^j$ in Algorithm S2 that is designed to ensure that we only aggregate over signal coordinates. The input parameters T^{diag} and T^{off} represent critical values for the diagonal and off-diagonal statistics S^{diag} and S^{off} respectively, both defined in these algorithms. In other words, we declare a change as soon as either $S^{\mathrm{diag}} \geq T^{\mathrm{diag}}$ or $S^{\mathrm{off}} \geq T^{\mathrm{off}}$.

Algorithm S1: Pseudo-code of the `ocd` algorithm

Input: $X_1, X_2, \ldots \in \mathbb{R}^p$ observed sequentially, $\beta > 0$, $\tilde{a} \geq 0$, $T^{\mathrm{diag}} > 0$ and $T^{\mathrm{off}} > 0$

Set: $b_{\min} = \frac{\beta}{\sqrt{2^{\lfloor \log_2(2p) \rfloor} \log_2(2p)}}$, $\mathcal{B}_0 = \{\pm b_{\min}\}$,
$\mathcal{B} = \bigl\{\pm 2^{m/2} b_{\min} : m = 1, \ldots, \lfloor \log_2(2p) \rfloor \bigr\}$, $n = 0$, $A_b = \mathbf{0} \in \mathbb{R}^{p \times p}$ and
$t_b = 0 \in \mathbb{R}^p$ for all $b \in \mathcal{B} \cup \mathcal{B}_0$

repeat

  $n \leftarrow n + 1$

  observe new data vector X_n

  for $(j, b) \in [p] \times (\mathcal{B} \cup \mathcal{B}_0)$ **do**

    $t_b^j \leftarrow t_b^j + 1$

    $A_b^{\cdot,j} \leftarrow A_b^{\cdot,j} + X_n$

    if $bA_b^{j,j} - b^2 t_b^j / 2 \leq 0$ **then**

      $t_b^j \leftarrow 0$ and $A_b^{\cdot,j} \leftarrow 0$

    compute $Q_b^j \leftarrow \sum_{j' \in [p] : j' \neq j} \frac{(A_b^{j',j})^2}{t_b^j \vee 1} \mathbb{1}_{\bigl\{|A_b^{j',j}| \geq \tilde{a} \sqrt{t_b^j}\bigr\}}$

  $S^{\mathrm{diag}} \leftarrow \max_{(j,b) \in [p] \times (\mathcal{B} \cup \mathcal{B}_0)} \bigl(bA_b^{j,j} - b^2 t_b^j / 2 \bigr)$

  $S^{\mathrm{off}} \leftarrow \max_{(j,b) \in [p] \times \mathcal{B}} Q_b^j$

until $S^{\mathrm{diag}} \geq T^{\mathrm{diag}}$ or $S^{\mathrm{off}} \geq T^{\mathrm{off}}$;

Output: $N = n$

Algorithm S2: Pseudo-code for the `ocd′` algorithm, a slight variant of `ocd`

Input: $X_1, X_2, \ldots \in \mathbb{R}^p$ observed sequentially, $\beta > 0$, $\tilde{a} \geq 0$, $T^{\mathrm{diag}} > 0$ and $T^{\mathrm{off}} > 0$

Set: $b_{\min} = \frac{\beta}{\sqrt{2^{\lfloor \log_2(2p) \rfloor} \log_2(2p)}}$, $\mathcal{B}_0 = \{\pm b_{\min}\}$,

$\mathcal{B} = \bigl\{\pm 2^{m/2} b_{\min} : m = 1, \ldots, \lfloor \log_2(2p) \rfloor\bigr\}$, $n = 0$, $A_b = \Lambda_b = \tilde{\Lambda}_b = \mathbf{0} \in \mathbb{R}^{p \times p}$

and $t_b = \tau_b = \tilde{\tau}_b = 0 \in \mathbb{R}^p$ for all $b \in \mathcal{B} \cup \mathcal{B}_0$

repeat

- $n \leftarrow n + 1$
- observe new data vector X_n
- **for** $(j, b) \in [p] \times (\mathcal{B} \cup \mathcal{B}_0)$ **do**
  - $t_b^j \leftarrow t_b^j + 1$ and $A_b^{\cdot,j} \leftarrow A_b^{\cdot,j} + X_n$
  - set $\delta = 0$ if t_b^j is a power of 2 and $\delta = 1$ otherwise.
  - $\tau_b^j \leftarrow \tau_b^j \delta + \tilde{\tau}_b^j (1 - \delta) + 1$ and $\Lambda_b^{\cdot,j} \leftarrow \Lambda_b^{\cdot,j} \delta + \tilde{\Lambda}_b^{\cdot,j} (1 - \delta) + X_n$
  - $\tilde{\tau}_b^j \leftarrow (\tilde{\tau}_b^j + 1)\delta$ and $\tilde{\Lambda}_b^{\cdot,j} \leftarrow (\tilde{\Lambda}_b^{\cdot,j} + X_n)\delta$.
  - **if** $b A_b^{j,j} - b^2 t_b^j / 2 \leq 0$ **then**
    - $t_b^j \leftarrow \tau_b^j \leftarrow \tilde{\tau}_b^j \leftarrow 0$
    - $A_b^{\cdot,j} \leftarrow \Lambda_b^{\cdot,j} \leftarrow \tilde{\Lambda}_b^{\cdot,j} \leftarrow 0$
  - $\Xi_b^{\cdot,j} \leftarrow \Lambda_b^{\cdot,j} / (\tau_b^j \vee 1)^{1/2}$
  - $\tilde{Q}_b^j \leftarrow \sum_{j' \in [p] \setminus \{j\}} (\Xi_b^{j',j})^2 \mathbb{1}_{\{|\Xi_b^{j',j}| \geq \tilde{a}\}}$
- $S^{\mathrm{diag}} \leftarrow \max_{(j,b) \in [p] \times (\mathcal{B} \cup \mathcal{B}_0)} \bigl(b A_b^{j,j} - b^2 t_b^j / 2\bigr)$
- $S^{\mathrm{off}} \leftarrow \max_{(j,b) \in [p] \times \mathcal{B}} \tilde{Q}_b^j$

until $S^{\mathrm{diag}} \geq T^{\mathrm{diag}}$ or $S^{\mathrm{off}} \geq T^{\mathrm{off}}$;

Output: $N = n$

S4. RESULTS UNDER SUB-GAUSSIAN AND SUB-EXPONENTIAL ASSUMPTIONS

This section provides justification for the claimed theoretical results in Section 3.4 of the main text. We will rely on the following three propositions, the first of which is standard (e.g. Wainwright 2019, Proposition 2.5 and (2.18)).

Proposition S5 (Hoeffding-type and Bernstein-type tail bound)**.** *(a) Let $a_1, \ldots, a_n \in \mathbb{R}$ and let $X_1, \ldots, X_n$ be independent sub-Gaussian random variables with variance parameter 1. Then*

$$\mathbb{P}\Big(\sum_{i=1}^{n} a_i X_i \geq x\Big) \leq \exp\biggl(-\frac{x^2}{2\sum_{i=1}^{n} a_i^2}\biggr)$$

for all $x \geq 0$.

(b) Let $a_1, \ldots, a_n \in \mathbb{R}$ and let $X_1, \ldots, X_n$ be independent sub-exponential random variables with variance parameter 1 and rate parameter $A > 0$. Then

$$\mathbb{P}\Big(\sum_{i=1}^{n} a_i X_i \geq x\Big) \leq \exp\biggl\{-\min\biggl(\frac{x^2}{2\sum_{i=1}^{n} a_i^2}, \frac{Ax}{2\max_{i\in[n]}|a_i|}\biggr)\biggr\}$$

for all $x \geq 0$.

One special case where we apply this proposition frequently is with $a_1 = \ldots = a_n = n^{-1/2}$. The two bounds are then $e^{-x^2/2}$ and $e^{-x(x\wedge A\sqrt{n})/2}$ respectively.

The following proposition can be used in place of Lemma S2 to control excursion probabilities of sub-Gaussian and sub-exponential random walks with drift.

Proposition S6. *Let $\mu \in \mathbb{R}$ and let $Y_1, Y_2, \ldots$ be independent random variables. Define $U_n := \sum_{i=1}^{n} Y_i$ for $n \in \mathbb{N}$ with $U_0 := 0$, and let $\xi := \operatorname{sargmin}_{n\in\mathbb{N}_0} \mu U_n$.*

(a) Assume that $Y_1 - \mu, Y_2 - \mu, \ldots$ are independent sub-Gaussian random variables with variance parameter 1. Then

$$\mathbb{P}(\xi \geq y) \leq \mathbb{P}\Big(\inf_{n \in \mathbb{N}_0 : n \geq y} \mu U_n \leq 0\Big) \leq 3(\mu^{-2} \vee 1) e^{-y\mu^2/2}$$

for $y \in [0, \infty)$.

(b) Assume that $Y_1 - \mu, Y_2 - \mu, \ldots$ are independent sub-exponential random variables with variance parameter 1 and rate parameter $A > 0$. Then

$$\mathbb{P}(\xi \geq y) \leq \mathbb{P}\Big(\inf_{n \in \mathbb{N}_0 : n \geq y} \mu U_n \leq 0\Big) \leq 3\Big(\frac{1}{\mu^2} \vee \frac{1}{\mu A} \vee 1\Big) e^{-y\mu(\mu \wedge A)/2}$$

for $y \in [0, \infty)$.

Proof. Following the same argument at the beginning of the proof of Lemma S2, it suffices to only prove the latter inequality for both results for $\mu > 0$.

(a) By a union bound and Proposition S5(a), we have

$$\begin{aligned}\mathbb{P}\Big(\inf_{n \in \mathbb{N}_0 : n \geq y} \mu U_n \leq 0\Big) &\leq \sum_{n=\lceil y \rceil}^{\infty} \mathbb{P}(U_n \leq 0) = \sum_{n=\lceil y \rceil}^{\infty} \mathbb{P}(U_n - n\mu \leq -n\mu) \leq \sum_{n=\lceil y \rceil}^{\infty} e^{-n\mu^2/2} \\ &\leq \frac{e^{-y\mu^2/2}}{1 - e^{-\mu^2/2}} \leq \frac{e^{-y\mu^2/2}}{\frac{\mu^2}{4\log 2} \wedge \frac{1}{2}} \leq 3(\mu^{-2} \vee 1) e^{-y\mu^2/2}.\end{aligned}$$

(b) Again, by a union bound and Proposition S5(b), we have

$$\begin{aligned}\mathbb{P}\Big(\inf_{n \in \mathbb{N}_0 : n \geq y} \mu U_n \leq 0\Big) &\leq \sum_{n=\lceil y \rceil}^{\infty} \mathbb{P}(U_n - n\mu \leq -n\mu) \leq \sum_{n=\lceil y \rceil}^{\infty} e^{-n\mu(\mu \wedge A)/2} \\ &\leq 3\Big(\frac{1}{\mu^2} \vee \frac{1}{\mu A} \vee 1\Big) e^{-y\mu(\mu \wedge A)/2},\end{aligned}$$

where the last inequality follows from the last three inequalities in the proof for the (a) part, with $\mu(\mu \wedge A)$ taking the place of μ^2. $\square$

Our final preparatory result will be used to modify (S17) in the proof of Proposition 5, which can be traced back to the proof of Chen et al. (2022, Theorem 1). Let $b \neq 0$, $T^{\mathrm{diag}} > 0$ and let $Z_1, Z_2, \ldots$ be independent centered random variables. We define the stopping time

$$N_{\mathrm{os}} := \inf\Big\{n \in \mathbb{N} : b\sum_{t=1}^{n}(Z_t - b/2) \geq T^{\mathrm{diag}}\Big\}, \tag{S37}$$

where 'os' stands for one-sided. When $Z_1, Z_2 \ldots \overset{\mathrm{iid}}{\sim} N(0,1)$, we can use a sequential probability ratio test argument to show that $\mathbb{P}(N_{\mathrm{os}} < \infty) \leq e^{-T^{\mathrm{diag}}}$. The aim of Proposition S7 below is to establish similar bounds under the sub-Gaussian and the sub-exponential distributional assumptions respectively.

Proposition S7. *(a) Let $b \neq 0$, $T^{\mathrm{diag}} > 0$ and let $Z_1, Z_2, \ldots$ be independent sub-Gaussian random variables each with variance parameter* 1*. Then N_{os} defined in* (S37) *satisfies*

$$\mathbb{P}(N_{\mathrm{os}} < \infty) \leq e^{-T^{\mathrm{diag}}}.$$

(b) Let $T^{\mathrm{diag}} > 0$ and $Z_1, Z_2, \ldots$ be independent sub-exponential random variables with variance parameter 1 *and rate parameter $A > 0$. Let $b \in [-A, A] \setminus \{0\}$. Then*

$$\mathbb{P}(N_{\mathrm{os}} < \infty) \leq e^{-T^{\mathrm{diag}}}.$$

Proof. Without loss of generality, assume $b > 0$ in part (a) and $0 < b < A$ in part (b). The following argument then holds for both cases. Denote $S_n := \sum_{t=1}^{n} Z_t$ with $S_0 := 0$, $V_n := \exp\{(bS_n - b^2 n/2) \wedge T^{\mathrm{diag}}\}$ and $\mathcal{F}_n = \sigma(Z_1, \ldots, Z_n)$ with $\mathcal{F}_0$ defined to be trivial

σ-algebra. Then for $n \in \mathbb{N}$, we have

$$\begin{aligned}\mathbb{E}[V_n \mid \mathcal{F}_{n-1}] &= \mathbb{E}\big[V_n \mathbb{1}_{\{V_{n-1} < e^{T^{\mathrm{diag}}}\}} \bigm| \mathcal{F}_{n-1}\big] + \mathbb{E}\big[V_n \mathbb{1}_{\{V_{n-1} = e^{T^{\mathrm{diag}}}\}} \bigm| \mathcal{F}_{n-1}\big] \\ &\leq \mathbb{E}\big[V_{n-1} e^{bZ_n - b^2/2} \mathbb{1}_{\{V_{n-1} < e^{T^{\mathrm{diag}}}\}} \bigm| \mathcal{F}_{n-1}\big] + \mathbb{E}\big[e^{T^{\mathrm{diag}}} \mathbb{1}_{\{V_{n-1} = e^{T^{\mathrm{diag}}}\}} \bigm| \mathcal{F}_{n-1}\big] \\ &\leq V_{n-1} \mathbb{1}_{\{V_{n-1} < e^{T^{\mathrm{diag}}}\}} + e^{T^{\mathrm{diag}}} \mathbb{1}_{\{V_{n-1} = e^{T^{\mathrm{diag}}}\}} = V_{n-1}.\end{aligned}$$

Hence $(V_n)_{n \geq 0}$ is a supermartingale with respect to the filtration $(\mathcal{F}_n)_{n \geq 0}$. Since $|V_n| \leq e^{T^{\mathrm{diag}}}$ for all n, we have by the Optional Stopping Theorem that

$$1 = \mathbb{E}V_0 \geq \mathbb{E}V_{N_{\mathrm{os}}} = \mathbb{E}\big[V_{N_{\mathrm{os}}} \mathbb{1}_{\{N_{\mathrm{os}} < \infty\}} + V_{N_{\mathrm{os}}} \mathbb{1}_{\{N_{\mathrm{os}} = \infty\}}\big] \geq e^{T^{\mathrm{diag}}} \mathbb{P}(N_{\mathrm{os}} < \infty),$$

as required. □

The modifications to the proofs of Theorems 1, 2 and 3, as well as Proposition 5 that are needed in the sub-Gaussian and sub-exponential settings are as follows. In (S1), (S5), (S6), (S8), (S9), (S10), (S11), (S19), (S30), (S33) and (S35), we apply Proposition S5 in place of the Gaussian tail bounds; in (S2), (S4) and (S26), we apply Proposition S6 in place of Lemma S2; and finally, we apply Proposition S7 to yield the bound corresponding to (S17).

S5. ADDITIONAL SIMULATION RESULTS

Table S1 provides additional simulation results for the `ocd_CI` procedure under spatial dependence. The data generating mechanisms and conclusions from these results are given in Section 4.2 of the main text.

Table S1: Spatial dependence. Estimated coverage and average length of the `ocd_CI` confidence interval and average detection delay over 2000 repetitions, with standard errors in brackets, under a Toeplitz cross-sectional covariance matrix Σ with entries $\Sigma_{jk} = \rho^{|j-k|}$ for $j, k \in [p]$. Other parameters: $p = 100$, $\beta = \vartheta$, $\gamma = 30000$, $z = 1000$, $\alpha = 0.05$, $a = \tilde{a} = \sqrt{2 \log p}$, $c = 0.5$, $d_1 = c\sqrt{\log(p/\alpha)}$, $d_2 = 4d_1^2$.

ρ	s	ϑ	Detection Delay	Coverage (%)	CI Length
0.5	2	2	$13.9_{(0.1)}$	$98.5_{(0.3)}$	$35.5_{(1.0)}$
0.5	2	1	$49.1_{(0.3)}$	$99.0_{(0.2)}$	$125.1_{(1.6)}$
0.5	2	0.5	$172.5_{(1.0)}$	$99.5_{(0.2)}$	$447.0_{(2.8)}$
0.5	10	2	$21.9_{(0.1)}$	$98.7_{(0.3)}$	$42.0_{(0.9)}$
0.5	10	1	$76.1_{(0.5)}$	$98.8_{(0.2)}$	$154.2_{(1.5)}$
0.5	10	0.5	$266.7_{(1.8)}$	$99.0_{(0.2)}$	$566.9_{(3.9)}$
0.5	100	2	$52.1_{(0.3)}$	$98.3_{(0.3)}$	$106.8_{(0.9)}$
0.5	100	1	$187.7_{(1.3)}$	$98.4_{(0.3)}$	$399.5_{(3.3)}$
0.5	100	0.5	$655.3_{(5.0)}$	$98.5_{(0.3)}$	$1366.2_{(10.1)}$
0.75	2	2	$13.9_{(0.1)}$	$96.9_{(0.4)}$	$51.1_{(2.6)}$
0.75	2	1	$47.9_{(0.3)}$	$96.8_{(0.4)}$	$146.0_{(3.3)}$
0.75	2	0.5	$171.5_{(1.1)}$	$97.7_{(0.3)}$	$463.8_{(4.2)}$
0.75	10	2	$21.8_{(0.2)}$	$96.4_{(0.4)}$	$48.6_{(1.7)}$
0.75	10	1	$75.3_{(0.5)}$	$96.7_{(0.4)}$	$165.0_{(2.7)}$
0.75	10	0.5	$266.3_{(1.9)}$	$96.0_{(0.4)}$	$558.9_{(4.5)}$
0.75	100	2	$50.9_{(0.3)}$	$96.8_{(0.4)}$	$106.8_{(1.2)}$
0.75	100	1	$184.8_{(1.4)}$	$95.6_{(0.5)}$	$401.8_{(3.8)}$
0.75	100	0.5	$647.3_{(5.4)}$	$94.6_{(0.5)}$	$1312.3_{(11.2)}$

References

Chen, Y., Wang, T., and Samworth, R. J. (2022), "High-dimensional, Multiscale Online Changepoint Detection," *J. Roy. Statist. Soc., Ser. B*, 84, 234–266.

Massart, P. (2007), *Concentration inequalities and model selection: Ecole d'Eté de Probabilités de Saint-Flour XXXIII-2003*, Springer.

Siegmund, D. (1986), "Boundary Crossing Probabilities and Statistical Applications," *Ann. Statist.*, 14, 361–404.

Wainwright, M. (2019), *High-dimensional Statistics: A Non-asymptotic Viewpoint*, Cambridge: Cambridge University Press.

Yu, B. (1997), "Assouad, Fano and Le Cam," in D. Pollard, E. Torgersen, and Y. G. L. (eds.), "Festschrift for Lucien Le Cam: Research Papers in Probability and Statistics," New York: Springer, pp. 423–435.
